# Factors Associated with National Health Insurance Coverage in Indonesia

**DOI:** 10.12688/f1000research.53672.1

**Published:** 2021-07-14

**Authors:** Tintin Sukartini, Hidayat Arifin, Yulia Kurniawati, Rifky Octavia Pradipta, Nursalam Nursalam, Joel Rey Ugsang Acob

**Affiliations:** 1 Department of Advanced Nursing, Faculty of Nursing, Universitas Airlangga, Surabaya, Indonesia; 2 Department of Medical-Surgical Nursing, Faculty of Nursing, Universitas Padjadjaran, Bandung, Indonesia; 3 Visayas State University, City of Baybay, Philippines

**Keywords:** health insurance; health policy; demographic health survey; Indonesia

## Abstract

**Background: **The National Health Insurance (NHI) program is the Indonesian government's national health program. However, health insurance coverage has not been maximized. This study aims to analyze the factors associated with health insurance coverage in Indonesia.

**Methods: **Cross-sectional data were obtained from the Indonesian Demographic and Health Survey 2017. A total of 39,580 respondents were selected using two-stage stratified cluster sampling. The data come from the DHS Questionnaire Phase 7. The variables include age, education level, wealth quintiles, residence, the number of children who are alive, marital status, current employment status, earnings, and health insurance status. The data were analyzed using chi-squared and binary logistic analyses.

**Results:** The prevalence of health insurance coverage in the Indonesian population is 62.3%. Respondent age [AOR=0.875; 95% CI=0.763-0.967], education level [AOR=0.437; 95% CI=0.410-0.466], wealth quintile [AOR=0.762; 95% CI=0.713-0.815], residence [AOR=0.762; 95% CI=0.713-0.815], marital status [AOR=0.718; 95% CI=0.625-0.825] and earnings [AOR=1.245; 95% CI=1.177-1.316] are related to health insurance coverage. However, the number of children who are alive and respondents who were currently working were not significantly related to health insurance coverage in Indonesia.

**Conclusion:** The government needs to pay attention to the characteristics and demographic conditions of the Indonesian population to be able to achieve maximum health insurance coverage. Maximum coverage through the provision of appropriate information should be promoted.

## Introduction

The National Health Insurance (NHI) is the Indonesian government's program which provides people with the chance to access health services for health promotion, illness prevention, illness treatment, and rehabilitation at an affordable cost. ^1, 2^ The Indonesian government started the NHI program in 2004, provided in the form of the Social Security Administrator (SSA) which is divided into two sectors namely the Social Security Administrator for Health (SSAH) and the Social Security Administrator for Employment (SSAE). However, the full coverage target was not achieved in 2017. ^3^ Another problem remains, namely, that the administrative system of the SSAH in Indonesia, which is related to health care services, lacks a sufficient quantity of essential care offices of reasonable quality. This is in addition to inadequate access to explicit medications and clinical supplies, the mistargeting of low-income and middle income populations, the issue of inappropriate behavior, and unforeseen weak data frameworks. ^4^ These problems hamper the participation of the Indonesian people in NHI, meaning that full coverage is difficult to achieve.

Out of a population of approximately 267.3 million people, approximately 25.1 million Indonesians live below the poverty line. According to the data from March 2019, approximately 20.6% of the whole population is powerless to prevent falling into neediness, as their income barely drifts over the national poverty line. ^5^ In the most recent decade, the prevalence of needy individuals in Indonesia declined from 19 to 11%. However, the malnutrition rate shows no significant reduction. ^6^ Indonesia has encountered a twofold ailment problem wherein the frequency of noncommunicable diseases (NCDs) is growing against a background of significant transmissible ailments, for example, tuberculosis and malaria. ^7^

There have been several previous studies regarding health insurance coverage. These studies have analyzed some of the determinants of insurance coverage, such as knowledge, ^8– 12^ cost, ^8, 12^ attitude and family support, ^9, 11, 13, 14^ age ^14, 15^ region, history of chronic disease, economic status, residency, ^15^ ability to pay, ^13, 16^ willingness to pay, average monthly expenses, ^13^ risk aversion, amount of loss, income, ^16, 17^ information, ^11, 18, 19^ religiosity, beliefs, ^18^ education, ^10, 15^ income, ^10, 11^ motivation, intention, ^10^ institutional policies, ^17^ perception, ^10, 19^ social support, ^19^ distance, and socialization. ^12, 20^ Therefore, this study presents new findings that determine the importance of NHI factors consisting of wealth quintile, residence, number of living children, marital status, current employment status and earnings.

The NHI aims to ensure that all Indonesian citizens have access to health services, especially the poor and near-poor. The development of the health service sector in Indonesia provides an opportunity for the Indonesian government to succeed in this program. In 2017, five provinces managed to achieve universal NHI coverage, which mostly included large cities in Indonesia, such as West Java (5.59%), Central Java (4.07%), Aceh (4.01%), East Java (3.87%), and North Sumatra (2.91%). ^21^ The larger the number of residents and the demographic location, the greater the achievement in NHI coverage. In the eastern part of Indonesia, the NHI coverage is lower. Since 2014, when NHI was initiated, Indonesia has made steady progress, with approximately 133,423,653 people becoming members of the NHI, but this is still far from Indonesia’s total population of 255,18 million people. ^22, 23^ This is due to demographic factors and the fact that Indonesia consists of islands or regions, which causes the uneven distribution of NHI coverage. It has been assessed that 34% of the population is uncovered, and a large portion of these are individuals working in the informal sector, such as beggars, farmers, breeders, and day laborers. The growth in membership among this group has continuously slowed, dropping from 6.55% per month (2015) to 2.17% per month (2016). ^24^ In light of these related issues, this investigation aims to understand the details of medical coverage inclusion and to deconstruct its determinant factors. The foundational attributes—for example, demographic characteristics, household characteristics, and financial condition—were selected for examination based on past investigations with certain adjustments due to information accessibility. This study aims to examine the determinants of health insurance coverage in Indonesia.

## Methods

### Study design

 This study uses a cross-sectional design. Data were obtained from the secondary data of the Indonesian Demographic and Health Survey (IDHS) 2017. IDHS, in obtaining the data, worked closely with Indonesian stakeholders and collaborated with the Inner-City Fund (ICF) International.

### Setting

**Figure 1.**
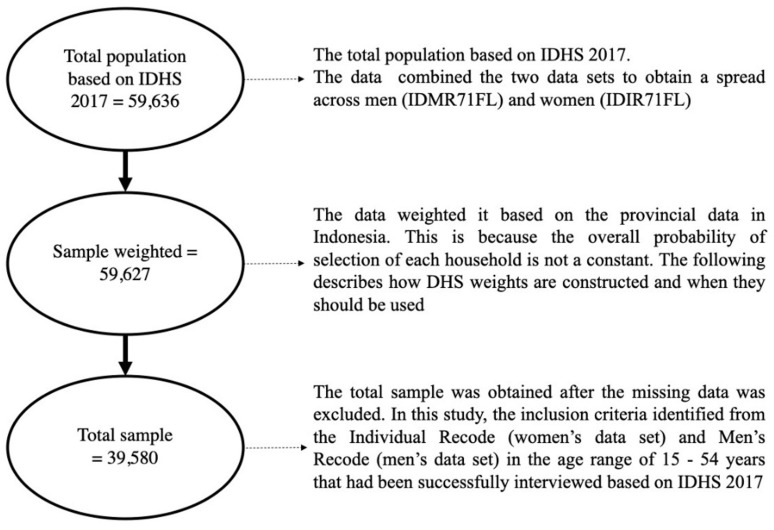
Study Sample Selection.

 This study uses data from IDHS 2017, which was conducted in December 2017. The study used the IDIR71FL (Indonesian Individual Recode phase 7) and IDMR71FL (Indonesian Men Recode phase 7) data sets. The data sets provide information about men and women within the age range of 15–54 years. For this study, the researchers combined the two data sets to obtain a total sample including both men and women. The sampling technique used by the IDHS was two-stage stratified cluster sampling, which includes selecting clusters from each stratum and a list of households in the selected clusters. Selected households were then interviewed by the IDHS. ^21^ The total sample was 59,636 respondents. Then, the researchers weighted the data based on the provincial data in Indonesia and obtained 59,627. This is because the overall probability of selection for each household is not constant. The following describes how DHS weights are constructed and when they should be used. ^25^ In this study, the inclusion criteria include being identified in the Individual Recode (women’s data set) or Men’s Recode (men’s data set), being in the age range of 15–54 years old, and having been successfully interviewed for the IDHS 2017. The exclusion criterion was having missing data. After excluding observations with missing data, the total sample size was 39,580 (see Figure 1).

### Variables

 The independent variables in the study are age, education level, wealth quintile, residence, number of living children, marital status, current employment status and earnings. The age variable is divided into categories: 15–24 years, 25–34 years old, 35–49 years old, and 50–54 years old. ^26^ Educational level is divided into four categories: high education, secondary education, primary education, and no education. ^27^ Wealth quintiles were measured using principal component analysis (PCA). ^28^ The categorization of the wealth quintiles includes richest, richer, middle, poorer, and poorest. ^29, 30^ The residence variable in this study contains the categories rural and urban. ^31^ The variable measuring the number of children who are alive is categorized into three categories namely, 0–4, 5–9, and 10–14. ^21^ The marital status variable is divided into six categories: single, married, with partner, widowed, divorced, and separated. ^21^ The current employment status variable is divided into two categories, namely, “yes” (currently working) and “no” (not currently working). ^21^ The variable for earnings is categorized into “not paid” if the respondent does not have an income and does not work. Meanwhile, the category of “paid” participants includes those who have an income in the form of cash only, cash and in-kind payments, or in-kind payments only. ^21^

 The dependent variable in this study is the coverage of the National Health Insurance, provided by the Indonesian government in the form of SSAH. The government states that all Indonesian citizens are required to become members of the NHI program with Indonesian Health Cards managed by the Indonesian government. ^32^ The health insurance coverage variable is divided into two categories, namely, “yes” (has coverage) and “no” (does not have coverage). Respondents are said to have health insurance if at the time of the interview, they said that they have health insurance and could show a membership card. If at the time of the interview, there are family members who do not have health insurance, then they are not included in the category of having health insurance. ^21^

### Research instruments

 This study uses the questionnaire from DHS Phase 7. The model in the DHS questionnaire emphasizes flexibility and basic indicators for the respondents. Health insurance coverage is available in the Women and Men Questionnaire Topics. ^33^ Validity and reliability tests were conducted to decrease the rate of errors. The policies made by DHS for using the questionnaires were printed in the local language to help the respondents better understand the meaning of each question. ^34^

### Data analysis

To analyze the factors associated with health insurance coverage in Indonesia, the researchers used chi-squared analysis and binary logistics, conducted using Stata 16.1. Both variables were assessed using an OR with a 95% CI to examine the strength of the association and p<0.05 was chosen to indicate statistical significance. We used the STATA version 16.1: “A Software resource for statistical analysis and presentation of graphics (Stata, RRID:SCR_012763)”.

### Ethical considerations

This study sought ethical approval from the Ministry of Health of Indonesia. The author was approved to use the following survey datasets obtained from ICF International as part of the Demographic Health Survey program with AuthLetter number 144520. DHS has policies requiring the use of questionnaires that have been translated and printed in all the major local languages in which interviews are expected to take place and requires signed informed consent. ^34^ The dataset policy is available on the official website.

## Results

**Figure 2.**
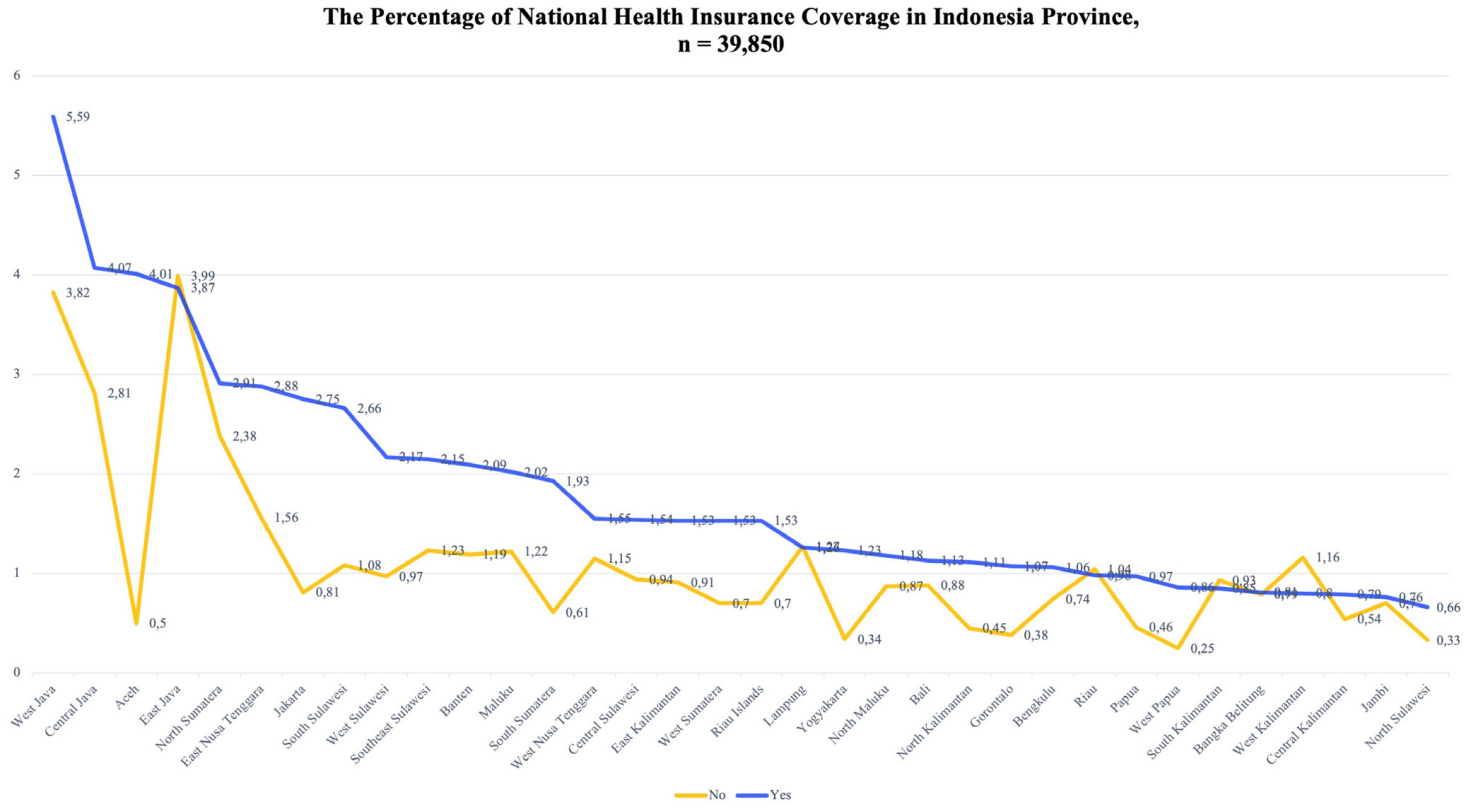
Distribution of National Health Insurance in Indonesia.

The achievements in NHI coverage are mostly found in large cities in Indonesia, such as West Java, Central Java, Aceh, East Java, and North Sumatra. This is also due to the large number of residents and the demographic characteristics of the population that allow for the achievement of greater NHI coverage. However, the increasing NHI coverage is still accompanied by people who are not enrolled in the NHI. When viewed proportionately, Aceh Province has a higher level of NHI coverage than other provinces. Indonesia is divided into three parts, namely, Western, Central and Eastern Indonesia. Traveling east, NHI coverage decreases. This could be due to the central government’s failure to make the NHI affordable. There are more provinces with a low level of NHI coverage than with a high level of coverage. Therefore, this should be a serious concern for the government ( Figure 2).

The prevalence of health insurance coverage in Indonesia is 62.3%. This still does not comply with government regulations that state that all Indonesian citizens are required to become members of the National Health Insurance-Indonesia Health Card program. The majority of respondents with health insurance were in the age range of 35–49 years (51.05%). The majority of respondents had a secondary school education (50.14%). Based on the wealth quintile data, many respondents were in the poorest category (22.09%), and the majority of residences were urban (52.69%). The data on the number of children who are alive showed that the majority were in the range 0 – 4 (94.39%). The majority of respondents were married (79.12%), currently working (92.17%), and had earnings (82.72%) ( Table 1).

**Table 1.** Respondent’s characteristics (n=39,580).

Characteristics	n	%
Health insurance coverage No Yes	14,922 24,658	37.7 62.3
Age (year) 50 – 54 35 – 49 25 – 34 15 – 24	1,443 20,207 11,458 6,472	3.65 51.05 28.95 16.35
Education level High education Secondary education Primary education No education	7,898 19,847 10,970 865	19.95 50.14 27.72 2.19
Wealth quintile Poorest Poorer Middle Richer Richest	8,745 7,318 7,508 7,770 8,239	22.09 18.49 18.97 19.63 20.82
Residence Urban Rural	20,856 18,724	52.69 47.31
The number of children who are alive 10 – 14 5 – 9 0 – 4	28 2,193 37,359	0.07 5.54 94.39
Marital status Single Married Partner Widowed Divorced Separated	5,909 31,316 286 784 1,153 132	14.93 79.12 0.72 1.98 2.91 0.33
Currently working No Yes	3,100 36,480	7.83 92.17
Respondent earnings No paid Paid	6,840 32,740	17.28 82.72

The results of the bivariate analysis show that all variables have a significant relationship with health insurance coverage in Indonesia, with p <0.001 ( Table 2).

**Table 2.** Bivariate analysis of the factors associated with health insurance coverage in Indonesia (n=39,580).

Variables	Health Insurance				X ^2^
	No		Yes		
	n	%	n	%	
Age (year) 50 – 54 35 – 49 25 – 34 15 – 24	532 7,331 4,484 2,575	1.34 18.52 11.33 6.51	911 12,876 6,974 3,897	2.30 32.53 17.62 9.85	39.81 ***
Education level High Secondary Primary No	1,722 7,931 4,895 374	4.35 20.04 12.37 0.94	6,176 11,916 6,075 491	15.60 30.11 15.35 1.24	1100.0 ***
Wealth quintile Poorest Poorer Middle Richer Richest	3,418 3,069 3,227 2,972 2,236	8.64 7.75 8.15 7.51 5.65	5,327 4,249 4,281 4,798 6,003	13.46 10.74 10.82 12.12 15.17	544.47 ***
Residence Urban Rural	7,090 7,832	17.91 19.79	13,766 10,892	34.78 27.52	257.78 ***
The number of children who are alive 10 – 14 5 – 9 0 – 4	6 644 14,272	0.02 1.63 36.06	22 1,549 23,087	0.06 3.91 58.33	72.01 ***
Marital status Single Married Partner Widowed Divorced Separated	2,166 11,736 131 291 531 67	5.47 29.65 0.33 0.74 1.34 0.17	3,743 19,580 155 493 622 65	9.46 49.47 0.39 1.25 1.57 0.16	55.36 ***
Currently working No Yes	1,263 13,659	3.19 34.51	1,837 22,821	4.64 57.66	13.24 ***
Respondent earnings Not paid Paid	3,059 11,863	7.73 29.97	3,781 20,877	9.55 52.75	173.56 ***

X2: chi-square; * p<0.1; ** p<0.05; *** p<0.01

The multivariate analysis shows that age, education level, wealth quintile, marital status, and earnings have very significant relationships with health insurance coverage. The odds of respondents aged 15–24 years old being covered were 0.875 times lower than those of the comparison group. [AOR=0.875; 95% CI=0.763-0.967]. The odds of respondents with a secondary education level being covered were 0.437 times lower than those of the comparison group [AOR=0.437; 95% CI=0.410-0.466]. Respondents in a poorer wealth quintile had odds of having health insurance that were 0.762 times lower than those of the comparison group [AOR=0.762; 95% CI=0.713-0.815]. Respondents who lived in a rural area had odds of being covered that were 0.782 times lower than those in urban areas [AOR=0.762; 95% CI=0.713-0.815]. Respondents with a marital status of divorced had odds of coverage that were 0.718 times lower than those of the comparison group [AOR=0.718; 95% CI=0.625-0.825]. The odds of respondents who received paid earnings being covered were approximately 1.245 times higher than those of respondents who were not paid [AOR=1.245; 95% CI=1.177-1.316]. However, the number of children who are alive and the current employment status variables were not significantly related to health insurance coverage in Indonesia ( Table 3).

**Table 3.** Multivariate analysis of the factors associated with health insurance coverage in Indonesia (n=39,580).

Variables	AOR	95% CI	
		Lower	Upper
Age (year) 50 – 54 35 – 49 25 – 34 15 – 24	1.000 1.094 0.859 ** 0.875 **	0.976 0.763 0.765	1.226 0.967 1.000
Education level High Secondary Primary No	1.000 0.437 *** 0.338 *** 0.330 ***	0.410 0.313 0.283	0.466 0.364 0.386
Wealth quintile Poorest Poorer Middle Richer Richest	1.000 0.762 *** 0.672 *** 0.714 *** 0.935 *	0.713 0.628 0.664 0.863	0.815 0.720 0.767 1.013
Residence Urban Rural	1.000 0.782 ***	0.745	0.821
The number of children who are alive 10 – 14 5 – 9 0 – 4	1.000 0.697 0.426 *	0.279 0.171	1.740 1.061
Marital status Single Married Partner Widowed Divorced Separated	1.000 1.068 * 0.813 1.028 0.718 *** 0.652 **	0.988 0.635 0.869 0.625 0.458	1.154 1.040 1.215 0.825 0.929
Currently working No Yes	1.000 1.018	0.942	1.100
Respondent earnings Not paid Paid	1.000 1.245 ***	1.177	1.316

X2: chi-square; * p<0.1; ** p<0.05; *** p<0.01; AOR: Adjusted Odd Ratio; CI: Confident Interval

## Discussion

The NHI target of Indonesia is was that all Indonesians must be insured by 2019. ^35^ It is important to know the factors associated with health insurance coverage so that a better and more appropriate policy can be developed. This study found that age, education level, wealth quintile, residence, marital status, and earnings type were associated with health insurance coverage.

Younger people are less likely to have health insurance. Old age increases the likelihood of health insurance enrollment and ownership. ^36– 38^ The older age groups were employed, enabling the purchase of insurance. ^39^ Younger citizens are less likely to have health insurance because they have low access to both employer-sponsored and self-financed health insurance. These age groups were dominated by those who were still in school and depend on their parents economically. Membership schemes and insurance financing that involve all family members in the insurance program can be applied to increase insurance coverage at a younger age. The government can use this scheme as a form of health insurance membership recruitment.

Having only a secondary education level or lower significantly decreases the likelihood of being insured. Citizens with secondary education or above have an increased likelihood of health insurance enrollment ^36^ and vice versa. ^38^ A secondary or high education level for women is also associated with an increased rate of health insurance coverage. ^40^ Education plays an important role in terms of imparting a level of knowledge and understanding about health insurance. Educated people are better at understanding the concept, benefits, and use of health insurance for the household, so they can make decisions about health insurance enrollment and understand its purpose of guarding them from sudden medical expenses. The government can promote health insurance programs in schools and companies. In addition to promotion at the school level targeting students and teachers, promotion at the company level targeting more varied levels of education: this way individuals with low to high levels of education can be easily reached.

The poorer, middle, richer, and richest wealth quintiles were less likely to have health insurance than poorest. The previous study stated that there was no significant association between wealth quintile and health insurance. ^41^ Another study stated that the richest households were more likely to have health insurance. ^36, 42, 43^ This could have occurred because the health insurance scheme in Indonesia is different from that of other countries. The poorest populations receive subsidized insurance to maintain and increase their health status, ^44^ such as payment for health services in the emergency department, ^45^ treatment for chronic illness, ^46^ and treatment of the factors associated with the success of diabetes mellitus management. ^47^ The poorest population’s health insurance is fully paid for by the government. The other schemes are paid for by the health insurance members themselves. This scheme makes the poorest populations more likely to be listed as health insurance member, making their coverage rate higher. The ownership of private health insurance may be the reason why the wealthier are less likely to have national health insurance. A study showed that the amount of monthly income as part of wealth is related to the demand for private insurance in Indonesia. ^48^ To increase the coverage of health insurance for other economic groups, the government can subsidize premiums for them. The government can work with companies to pass regulations stating that each company has an obligation to pay for insurance for its workers. This regulation can also protect the health of workers.

Rural households are less likely to have health insurance. ^40, 43^ The primary factor determining coverage for both the subsidized and contributory schemes in Indonesia is that citizens work and are urban residents of Java or Bali. ^49^ The affordability of travel to the health insurance office can be another reason for low coverage. The existence of branch offices in each city can increase the reach of insurance agencies. An increase in the number of insurance agencies can make it easier for potential participants to register for NHI. The government can support the spreading of branch offices by providing good infrastructure and communication networks like telephone and internet. The role of the government in providing infrastructure greatly affects the existence of branch offices and customer service.

Those who are divorced are less likely to have health insurance. Women often lose their health insurance in the months after a divorce ^50^ and thus become uninsured. ^51^ Divorced women are more likely to experience socioeconomic disadvantages than married women. ^52^ Divorced women lose their benefits in terms of health insurance as they are dropped from their husbands’ health insurance policies. They also lose their dependent payments and are unable to afford other forms of coverage. Jobless divorced woman find it difficult to pay monthly insurance premiums, so they decide to drop their insurance coverage.

Being employed or paid is likely to be associated with having health insurance. ^43^ Paid respondents are associated with the demand and possibility of having for health insurance enrollment. ^53, 54^ Paid employees obtain health insurance coverage from their employer. ^55^ The stable income of a paid employee also makes it easier for them to choose the health insurance agency appropriate to their needs. They have more flexibility in choosing their insurance class and the amount of their monthly premium. Companies usually have their employees become members and pays the monthly premium. To maintain and increase the coverage of health insurance for groups of paid employees, the government can work with employers by requiring them to provide their workers with insurance, and the premium is paid by the company.

## Implications and limitations

### Implications

 The results of this study can be used as basic information for the Indonesian government when determining the policies necessary to achieve maximum insurance coverage for the welfare of the community. The government can collaborate with employers to register and pay insurance premiums for employees, improve infrastructure and communication networks so as to increase the reach of insurance agency branch offices, maintain payment schemes for the poorest population, and provide subsidized insurance payments for the poor and the poorer.

### Limitations

This study used the data of men and women aged 15–54 years old. The government states that all residents must have health insurance. However, this study has not reviewed those under 15 years old or above 54 years old. In addition, other factors related to health insurance coverage can be studied for further information.

## Conclusion

Health is an important asset for the future. Early protection is needed even when the body is healthy so that there is protection and health insurance when you get sick. NHI helps Indonesian people to get health insurance in the future by providing easy access to health facilities for everyone. NHI coverage is influenced by several factors including age, education level, wealth quintiles, residence, the number of children who are alive, marital status, current employment status, earnings, and health insurance status. These factors cause not everyone agrees or even wishes to join the NHI program. The role of the government is needed to ensure that the NHI program is a solution to support health and access to quality services for everyone.

## Data availability

Indonesian Demographic Health Survey (IDHS) 2017 dataset is available online. Access to the dataset requires registration and is granted only for legitimate research purposes. A guide for how to apply for dataset access is available at: https://dhsprogram.com/data/Access-Instructions.cfm.
