# Supplementary material for: Factors Associated with National Health Insurance Coverage in Indonesia
Source: F1000Res. 2022 Sep 14;10:563. Originally published 2021 Jul 14. [Version 2] doi: 10.12688/f1000research.53672.2 (PMC9532955; doi:10.12688/f1000research.53672.2)
Supplement: Peer review history

# Peer review: Reviewer response for version 2

**DOI:** 10.5256/f1000research.138367.r150417

**Published:** 2022-10-04

**Authors:** Linlin Lindayani

**Affiliations:** 1 Sekolah Tinggi Ilmu Keperawatan PPNI Jawa Barat, Bandung, Indonesia

Thank you for the effort of authors to revise the paper.

Is the work clearly and accurately presented and does it cite the current literature?

Partly

If applicable, is the statistical analysis and its interpretation appropriate?

Yes

Are all the source data underlying the results available to ensure full reproducibility?

Yes

Is the study design appropriate and is the work technically sound?

Yes

Are the conclusions drawn adequately supported by the results?

Yes

Are sufficient details of methods and analysis provided to allow replication by others?

Yes

Reviewer Expertise:

Nursing technology, HIV, secondary analysis and review

I confirm that I have read this submission and believe that I have an appropriate level of expertise to confirm that it is of an acceptable scientific standard.

# Peer review: Reviewer response for version 1

**DOI:** 10.5256/f1000research.57086.r147665

**Published:** 2022-09-05

**Authors:** Mohd Khairul Zul Hasymi Firdaus

**Affiliations:** 1 Department of Medical Surgical Nursing, Kulliyyah (Faculty) of Nursing, International Islamic University Malaysia, Pahang, Malaysia

In general, this manuscript is interesting to read and contributes to a new body of knowledge. However, a little improvement may be required for this manuscript.

 Methods:

1. Study design: I would suggest including ‘retrospective’ in the study design since the authors use existing data from the 2017 survey. But if I am wrong, kindly ignore this suggestion.
2. Variable:
  - It is not clear how the authors define the variables, i.e. education level. It is suggested to use a similar term for ‘high educational’ level with other variables so that it will represent the similar attributes of the category. Maybe can use as a tertiary education level? And for ‘no education’, it is suggested to change it into 'no formal education'. so that it may cover both those who might receive informal education and those who never enrolled in the schooling system.
  - For the categorization of the wealth quintile, it is not clear how the authors define the poorest, poorer, middle, richer and richest. It would be good if the authors can explain further the categorisation of these words. It will help the reader to better understand this manuscript.
3. Research instrument: The authors mentioned that reliability and validity were conducted. It would be great if the authors can include the reliability and validity values of the used research instruments in this manuscript.

In terms of language, I would suggest for the authors to submit it to a proofreader so that it will be better.

 Other than that, it looks good.

Is the work clearly and accurately presented and does it cite the current literature?

Yes

If applicable, is the statistical analysis and its interpretation appropriate?

Yes

Are all the source data underlying the results available to ensure full reproducibility?

Yes

Is the study design appropriate and is the work technically sound?

Yes

Are the conclusions drawn adequately supported by the results?

Yes

Are sufficient details of methods and analysis provided to allow replication by others?

Yes

Reviewer Expertise:

NA

I confirm that I have read this submission and believe that I have an appropriate level of expertise to confirm that it is of an acceptable scientific standard.

# Response

**Published:** 2022-09-06

**Authors:** Hidayat Arifin

**Affiliations:** Universitas Padjadjaran, Indonesia

Dear Reviewer

 Thank your time to review our manuscript and for providing valuable comments and suggestions. We have pointed out all the issues. 

**Methods:**

 Study design: I would suggest including ‘retrospective’ in the study design since the authors use existing data from the 2017 survey. But if I am wrong, kindly ignore this suggestion.

***Response: **Thank you for your valuable suggestion. We have added the retrospective study design to the manuscript.*

**Variable:**

 It is not clear how the authors define the variables, i.e. education level. It is suggested to use a similar term for ‘high educational’ level with other variables so that it will represent the similar attributes of the category. Maybe can use as a tertiary education level? And for ‘no education’, it is suggested to change it into 'no formal education'. so that it may cover both those who might receive informal education and those who never enrolled in the schooling system.

***Response: **Thank you for your suggestion. The division of education categories was based on the Statute of the Republic of Indonesia.*

 For the categorization of the wealth quintile, it is not clear how the authors define the poorest, poorer, middle, richer and richest. It would be good if the authors can explain further the categorization of these words. It will help the reader to better understand this manuscript.

***Response:** Thank you, we elaborated more information about wealth quintiles in the methods sections.*

 Research instrument: The authors mentioned that reliability and validity were conducted. It would be great if the authors can include the reliability and validity values of the used research instruments in this manuscript.

***Response: **Thank you, we have mentioned the reliability and validity values*

 In terms of language, I would suggest for the authors submit it to a proofreader so that it will be better.

***Response:** Thank you, the manuscript was proofread by a professional.*

# Peer review: Reviewer response for version 1

**DOI:** 10.5256/f1000research.57086.r92728

**Published:** 2021-09-30

**Authors:** Linlin Lindayani

**Affiliations:** 1 Sekolah Tinggi Ilmu Keperawatan PPNI Jawa Barat, Bandung, Indonesia

This study provides important information on highlighting the factors associated with health insurance coverage in Indonesia. Since full coverage in Indonesia is difficult to achieve, this information will be very meaningful for the Indonesian government to ensure that the coverage of NHI programs could be increased by designing interventions targeted to the significant factors affected by the NHI coverage.

 However, I have few comments regarding this study:

**Abstract**

- The authors need to briefly describe the factors that will be explored and their significance.
- Please add information about the two-stage stratified cluster sampling, stratified by what?
- The government needs to pay attention to the characteristics and demographic conditions of the Indonesian population to be able to achieve maximum health insurance coverage. What kind of characteristics and demographic conditions of the Indonesian population? These should be pointed out according to the study findings.

**Introduction**

- The logic is quite confusing to address the research gap.
- There were some factors associated with low NHI coverage addressed from previous studies, such as belief and religiosity. Why did the authors not address these issues? Since Indonesia is one of the largest Muslim populations.
- Maybe some argument to just only focus on the demographic data (not because of the available data provided in the DHS Questionnaire).

**Methods**

- Since the data used IDHS 2017, researchers need justification that data from 2017 still has relevance with the current situations.
- Validity and reliability tests were conducted to decrease the rate of errors. Please mention the value of validity and reliability.

**Results**

- Be consistent in the tables -  use two decimals in each table and also in the text.

**Discussion**

- Important to highlight the low coverage of NHI.

Is the work clearly and accurately presented and does it cite the current literature?

Partly

If applicable, is the statistical analysis and its interpretation appropriate?

Yes

Are all the source data underlying the results available to ensure full reproducibility?

Yes

Is the study design appropriate and is the work technically sound?

Yes

Are the conclusions drawn adequately supported by the results?

Yes

Are sufficient details of methods and analysis provided to allow replication by others?

Yes

Reviewer Expertise:

Nursing technology, HIV, secondary analysis and review

I confirm that I have read this submission and believe that I have an appropriate level of expertise to confirm that it is of an acceptable scientific standard, however I have significant reservations, as outlined above.

# Response

**Published:** 2022-09-06

**Authors:** Hidayat Arifin

**Affiliations:** Universitas Padjadjaran, Indonesia

Dear Reviewer, 

 Thank you for the valuable suggestions and comments. Here we pointed out the comments and make a revision as suggested. 

**Abstract**

 The authors need to briefly describe the factors that will be explored and their significance.

***Response: **Thank you, we have described the factors that are explored in the methods sections.*

 Please add information about the two-stage stratified cluster sampling, stratified by what?

***Response: **Thank you, it was stratified by household. Detailed information about the sampling technique was presented in the methods section.*

 The government needs to pay attention to the characteristics and demographic conditions of the Indonesian population to be able to achieve maximum health insurance coverage. What kind of characteristics and demographic conditions of the Indonesian population? These should be pointed out according to the study findings.

***Response: **Thank you for the suggestion and clarification. After review, we have made a revision in the conclusion section.*

**Introduction**

 There were some factors associated with low NHI coverage addressed in previous studies, such as belief and religiosity. Why did the authors not address these issues? Since Indonesia is one of the largest Muslim populations. Maybe some argument to just only focus on the demographic data (not because of the available data provided in the DHS Questionnaire).

***Response: **Thank you for your concerns. We faced limitations to perform belief and religiosity data in the analysis because the data in 2017 IDHS is not supported. Since the secondary data is from IDHS, all the information only presented demographic information. However, we put these concerns as a limitation. Thank you for your valuable suggestions.*

**Methods**

 Since the data used IDHS 2017, researchers need justification that data from 2017 still has relevance to the current situations.

***Response: **Thank you, we have made sure that all the data presented has relevance to the current situations.*

 Validity and reliability tests were conducted to decrease the rate of errors. Please mention the value of validity and reliability.

***Response:** Thank you, we have presented the value of Cronbach alpha.*

**Results**

 Be consistent in the tables - use two decimals in each table and also in the text.

***Response:** Thank you, revised.*

**Discussion**

 Important to highlight the low coverage of NHI.

***Response: **Thank you for your valuable suggestion. We have elaborated information about the low coverage of NHI in the discussion section.*
